# Right Atrial Pressure Is Associated With Outcomes in Patient With Cardiogenic Shock Receiving Acute Mechanical Circulatory Support

**DOI:** 10.3389/fcvm.2021.563853

**Published:** 2021-02-11

**Authors:** Carlos D. Davila, Michele Esposito, Colin S. Hirst, Kevin Morine, Lena Jorde, Sarah Newman, Vikram Paruchuri, Evan Whitehead, Katherine L. Thayer, Navin K. Kapur

**Affiliations:** ^1^The Cardiovascular Center at Tufts Medical Center, Tufts University School of Medicine, Boston, MA, United States; ^2^Massachusetts General Hospital and Harvard Medical School, Boston, MA, United States

**Keywords:** cardiogenic shock, right atrial pressure, acute mechanical circulatory support, myocardial infarction, heart failure

## Abstract

**Background:** We describe the association between longitudinal hemodynamic changes and clinical outcomes in patients with cardiogenic shock (CS) receiving acute mechanical circulatory support devices (AMCS) at a single center. We hypothesized that improved right atrial pressure is associated with better survival in CS.

**Methods:** Retrospective analysis of patients from Tufts Medical Center that received AMCS for CS. Baseline characteristics and invasive hemodynamics were collected, analyzed, and correlated against outcomes. Hemodynamics were recorded at different time intervals during index admission [pre-AMCS, 24 h after AMCS (post AMCS), and last available set of hemodynamics (final-AMCS)]. Logistic regression was performed to determine variables associated with in-hospital mortality.

**Results:** A total of 76 patients had longitudinal hemodynamics available. In hospital mortality occurred in 46% of the cohort. Mean baseline right atrial pressure (RAP) was significantly higher among non-survivors vs. survivors (19.5+6.6 vs. 16.4+5.3 mmHg). Change in right atrial pressure from baseline to before device removal (ΔRA:final AMCS—pre AMCS) was significantly different between survivors and non survivors (−6.5 ± 6.9 mmHg vs. −2.5 ± 6.2 mmHg *p* = 0.03). Unadjusted logistic regression revealed baseline RAP (OR: 1.1 95% CI: 1.0–1.2), 24 h post device implant RAP (OR: 1.3 95% CI: 1.1–1.4), and final RAP (OR: 1.3 95% CI: 1.1–1.5) to be significant predictors of in-hospital mortality. In a multivariate logistic regression baseline RAP was no longer significantly associated with mortality in the overall cohort, while 24 h (OR: 1.26 95% CI: 1.1–1.5) and final RAP (OR: 1.3 95% CI: 1.1–1.6) remained statistically significant.

**Conclusion:** We report a novel retrospective analysis of hemodynamic changes in patients with CS receiving AMCS. Our findings identify the potential importance of venous congestion as a prognostic marker of mortality. Furthermore, early decongestion or reduced RA pressure is associated with better survival in these critically ill CS patients. These observations suggest the need for further study in larger retrospective and prospective cohorts of patients with varying degrees of CS severity.

## Introduction

Cardiogenic shock (CS) is a complex hemodynamic and metabolic syndrome associated with high in-hospital morbidity and mortality. CS is defined by insufficient cardiac output to maintain multi-organ perfusion. The landmark SHould we emergently revascularize Occluded Coronaries for Cardiogenic shocK (SHOCK) trial defined CS associated with acute myocardial infarction (AMI) using clinical and hemodynamic criteria including a systolic blood pressure <90 mmHg or the use of medical therapy to maintain a blood pressure of at least 90 mmHg, evidence of low tissue perfusion, a cardiac index of <2.2 L/min/m^2^, and an elevated pulmonary capillary wedge pressure of 15 mmHg (1). These criteria have since been used to diagnose and guide management for all etiologies of CS. Over the past decade, the growing use of acute mechanical circulatory support (AMCS) devices for left and right heart support such as veno-arterial extracorporeal membrane oxygenation (VA-ECMO) and percutaneous left ventricular assist devices (pLVAD), such as Impella or TandemHeart pumps for CS has increased awareness of the important role that hemodynamics play in defining CS severity (2). Recent data suggest that elevated right- and left-heart filling pressures may be important determinants of clinical outcomes associated with heart failure (3, 4). Systemic congestion and evidence of right ventricular dysfunction are now recognized as strong predictors of poor outcomes in CS due to AMI or heart failure (5–7). Few studies have explored the impact of hemodynamic changes during hospitalization for CS and little is known as to whether these changes impact clinical outcomes (8). To address this gap in knowledge, we examined the relationship between serial hemodynamics, indices of congestion, and clinical outcomes. We hypothesized that improved right atrial pressure is associated with better survival in CS.

## Methods

### Study Population

Data were retrospectively collected from patients admitted to Tufts Medical Center with refractory CS managed with acute mechanical circulatory support devices from June 2014 to January 2019. Inclusion criteria were patients > 18 years of age who presented with clinical signs of CS requiring a left-sided Impella, Impella 5.0, VA-ECMO, or TandemHeart device for support. Cardiogenic shock was defined using clinical and/or hemodynamic criteria from the SHOCK trial. The clinical criteria were: (1) hypotension: systolic blood pressure <90 mmHg for 30 min before inotropes/vasopressors; (2) end-organ hypoperfusion: cool extremities, oliguria (<30 mL/h) or anuria, altered mental status; and (3) tachycardia >100 beats per minute and/or hemodynamic criteria; (1) cardiac index ≤ 2.2 Lmin/m^2^ and (2) pulmonary capillary wedge pressure ≥ 15 mmHg.

Patients supported with multiple devices simultaneously or in sequence were included in this study. Exclusion criteria included lack of at least one complete set of available hemodynamic data.

Patient characteristics including demographic, laboratory data, hemodynamics, echocardiographic and clinical course were obtained from the medical record. Hemodynamic variables derived from pulmonary artery catheters were collected and analyzed in a retrospective fashion. Hemodynamic variables comprised; right atrial pressure (RA), pulmonary capillary wedge pressure (PCWP), and cardiac index (CI). Moreover, markers of right ventricular dysfunction were calculated from available hemodynamics including RA/PCWP, and pulmonary artery pulsatility index (PAPi). Hemodynamics were recorded at different time intervals during index admission. Pre-specified time points were: (1) Pre-AMCS deployment, or first set of hemodynamics available before AMCS implant, (2) Post AMCS, which included available hemodynamics recorded within 24 h of AMCS implantation, and (3) Final-AMCS, or last available set of hemodynamics before either AMCS explant or death. Lastly, the difference (Δ) between pre-AMCS and final post-AMCS hemodynamics was calculated and correlated against outcomes by logistic regression. For this particular analysis the primary clinical outcome of interest was in hospital all-cause mortality and cohort was stratified based on survivor status independent of the device(s) received.

### Statistical Analysis

Categorical variables are presented as frequencies and percentages and were compared by Fisher's exact test. For continuous variables, results are presented as mean ± standard deviation (SD) and continuous baseline characteristics were compared between survivors and non-survivors using two sample independent *t*-tests. A paired student's *T*-test was used to compare hemodynamics at different time points. Logistic regression was performed to determine variables associated with in-hospital mortality. All variables that were significant in univariate analysis with *p* < 0.05 were then selected for multivariate analysis. Hosmer-Lemeshow testing was used to show goodness of fit. A *p* < 0.05 was accepted as statistically significant for all tests. Statistical analysis was performed using GraphPad Prism software (GraphPad Software, La Jolla, CA) and IBM SPSS Statistics software (IBM Corp, Armonk, NY).

## Results

Hemodynamic data were available for analysis in 76 out of 130 patients receiving AMCS for cardiogenic shock. Baseline characteristics of the study population are shown in Table 1. Mean age was 58.4 ± 13.5 years and mean LVEF was 21.2 ± 16%. VA-ECMO, Impella, TandemHeart, or IABP was used to support 38/76 (50%), 49/76 (64%), 1/76 (1%), and 30/76 (39%) of patients, respectively. Indications for AMCS were: post-acute myocardial infarction (37%), acute on chronic heart failure (45%), myocarditis (9%), post cardiotomy shock (8%), and other (1%). Mean duration of mechanical support was 5.5 ± 3.8 days. In hospital mortality occurred in 46% of the cohort (*n* = 35).

**Table 1:** Baseline demographics and characteristics.

	**Total cohort (*n* = 76)**	**Survivors *n* = 41 (54%)**	**Non-survivors *n* = 35 (46%)**	***P*-value**
Age (years)	58.4 ± 13.5	53.2 ± 14.9	64.4 ± 8.5	<0.001
Gender (% male)	63.2	68.3	57.1	0.32
Admission LV ejection fraction (%)	21.2 ± 16	19 ± 14.5	24.7 ± 17.5	0.13
Previous myocardial infarction (%)	36.8	39	34.3	0.67
Previous heart failure (%)	55.3	61	48.6	0.28
Hypertension (%)	59.2	53.7	65.7	0.29
Diabetes (%)	35.5	36.6	34.3	0.83
Chronic kidney disease (%)	26.3	22	31.4	0.36
Prior PCI (%)	38.2	41.5	34.3	0.53
Prior CABG (%)	14.5	12.2	17.1	0.55
Intra-aortic balloon pump (%)	52.6	56.1	48.6	0.52
Duration of support (days)	5.5 ± 3.8	4.9 ± 3.3	6.2 ± 4.3	0.15
Out-of-hospital arrest (%)	16	15	17.1	0.8
Number of pressors/Inotropes	1.7 ± 1.2	1.5 ± 1.1	2 ± 1.2	0.04
**Device Type**				
Impella, *n* (%)	49 (64)	27 (66)	22 (63)	–
VA-ECMO, *n* (%)	38 (50)	23 (56)	15 (43)	–
IABP, *n* (%)	30 (39)	13 (32)	17 (49)	–
Tandem heart, *n* (%)	1 (1)	1 (2)	0	–
Device combination: ECMO + Impella, *n* (%)	10 (13)	6 (14)	4 (9)	–
**Etiology of Shock**				
Acute myocardial infarction, *n* (%)	28 (37)	15 (37)	13 (37)	0.96
Acute on chronic heart failure, *n* (%)	34 (45)	17 (41)	17 (49)	0.53
Myocarditis, *n* (%)	7 (9)	4 (10)	3 (9)	0.86
Post-cardiotomy, *n* (%)	6 (8)	4 (10)	2 (6)	0.51
Other, *n* (%)	1 (1)	1 (2)	0	–

Hemodynamic and laboratory data are shown in Table 2. Compared to survivors, non-survivors had a higher blood urea nitrogen (BUN) and lower hemoglobin levels. Mean lactate was 4.1 + 4.1 mEq/L and not significantly different between survivors and non-survivors. Mean arterial pressure was 71.3 + 17.3 mmHg and was significantly lower among non-survivors compared to survivors (64.8 + 15.2 vs. 77.1 + 17.3, *p* = 0.003). Mean cardiac index was 1.9 + 0.6 L/min/m^2^ for the total cohort and not significantly different between survivors and non-survivors. Mean PCWP was 24 + 7.5 mmHg and also not significantly different between survivors and non-survivors. Mean pulmonary artery pressure was 31.6 + 9.1 mmHg and was not significantly different between survivors and non-survivors.

**Table 2:** Baseline hemodynamics and laboratory data.

	**Total cohort (*n* = 76)**	**Survivors *n* = 41 (54%)**	**Non-survivors *n* = 35 (46%)**	***P*-value**
Sodium (mEq/L)	136.7 ± 6.2	136.5 ± 5.8	136.9 ± 6.8	0.77
Blood urea nitrogen (mg/dL)	38 ± 26.1	30.8 ± 21.2	47.5 ± 29.1	0.007
Creatinine (mg/dL)	2 ± 1.2	1.7 ± 1	2.2 ± 1.3	0.08
White blood count (K/uL)	15 ± 7.3	14.5 ± 6.3	15.8 ± 8.5	0.47
Hemoglobin (g/dL)	11.2 ± 2.7	12.2 ± 2.6	9.9 ± 2.4	<0.001
Hematocrit (%)	33.8 ± 7.9	36.4 ± 7.8	30.5 ± 6.9	0.002
Platelets (K/uL)	170.5 ± 97.6	179.7 ± 95.5	158.5 ± 100.7	0.37
AST (IU/L)	1006 ± 2975	863 ± 3104	1222 ± 2820	0.65
Total bilirubin (mg/dL)	2.1 ± 1.7	1.7 ± 1.4	2.6 ± 2	0.04
Lactate (mEq/L)	4.1 ± 4.1	3.7 ± 3.5	4.7 ± 4.8	0.4
Plasma-free hemoglobin (mg/dL)	17.1 ± 16.3	25.5 ± 23.9	10.7 ± 4.9	0.27
Mean arterial pressure (mmHg)	71.3 ± 17.3	77.1 ± 17.3	64.8 ± 15.2	0.003
Heart rate (beats per minute)	95.4 ± 22.6	97.9 ± 23.4	92.8 ± 21.7	0.37
Right atrial pressure (mmHg)	17.9 ± 6.1	16.4 ± 5.3	19.5 ± 6.6	0.03
PA systolic pressure (mmHg)	44.1 ± 13.7	40.1 ± 10.6	49 ± 15.6	0.007
PA diastolic pressure (mmHg)	25.7 ± 7.6	24.5 ± 7.1	27.1 ± 8	0.16
PA mean pressure (mmHg)	31.6 ± 9.1	29.7 ± 7.9	33.8 ± 10.1	0.06
PCWP (mmHg)	24 ± 7.5	23.6 ± 7.1	24.4 ± 8	0.66
Cardiac output (L/min)	4.7 ± 5.2	3.7 ± 1.3	5.9 ± 7.6	0.12
Cardiac index (L/min/m^2^)	1.9 ± 0.6	1.8 ± 0.6	2.1 ± 0.6	0.08
SVR (dynes-sec/cm^5^)	1140 ± 498	1285 ± 543	922 ± 334	0.05
PA saturation (%)	50.3 ± 16.2	53.5 ± 16.7	46.6 ± 14.9	0.13
PAPi	1.1 ± 0.7	1.1 ± 0.6	1.2 ± 0.8	0.32
RA/PCWP ratio	0.8 ± 0.3	0.7 ± 0.2	0.8 ± 0.3	0.1

**Figure 1:**
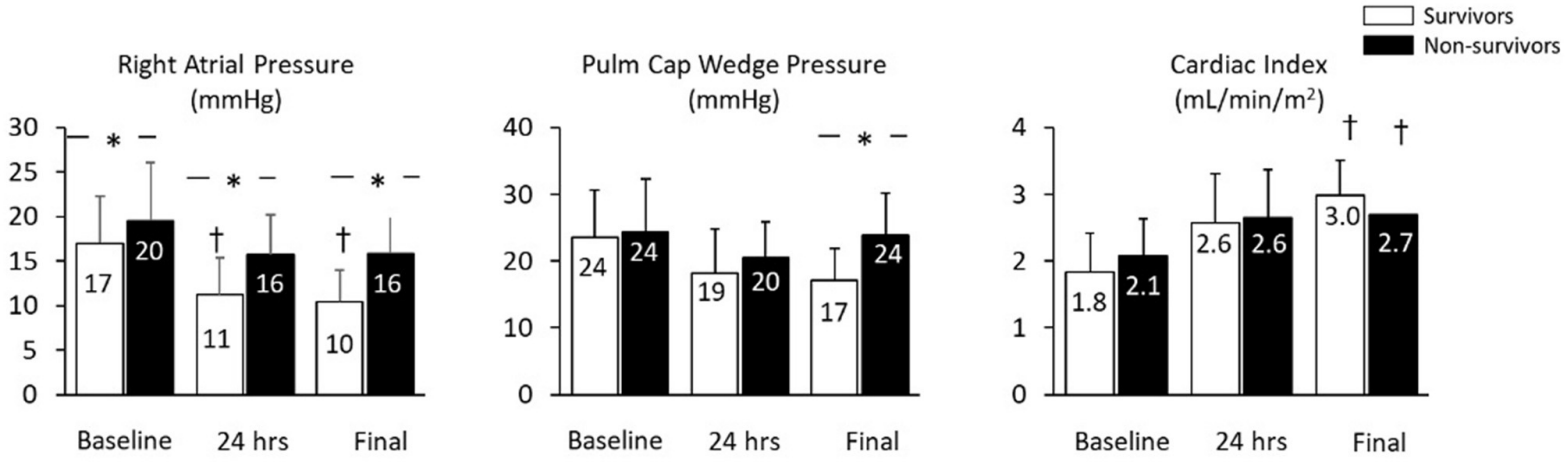
Different hemodynamic variables stratified by outcomes and time points.

Mean baseline right atrial pressure was 17.9 + 6.1 mmHg and was significantly higher among non-survivors (19.5 + 6.6 vs. 16.4 + 5.3 mmHg, *p* = 0.03). Compared to baseline values, mean right atrial pressure was lower within 24 h and before device removal among survivors, but was unchanged among non-survivors prior to device removal/death. At both 24 h and before device explant, mean right atrial pressure was lower among survivors compared to non-survivors (Figure 1). The change in right atrial pressure from baseline to before device removal (ΔRA:final AMCS—pre AMCS) was significantly different between survivors and non survivors (−6.5 ± 6.9 mmHg vs. −2.5 ± 6.2 mmHg *p* = 0.03). Unadjusted logistic regression revealed baseline RAP (OR 1.1 95% CI: 1.0–1.2), 24 h post device implant RAP (OR: 1.3 95% CI: 1.1–1.4), and final RAP (OR: 1.3 95% CI: 1.1–1.5) to be significant predictors of in-hospital mortality. However, change in RAP from baseline to the last collected was not a significant predictor of in-hospital mortality.

Mean PCWP was not significantly different between survivors and non-survivors at baseline or 24 h after initiation of AMCS. PCWP was higher among non-survivors at the time of explant or death. Cardiac Index (CI) at baseline, 24 h and at the final data recording timepoint was not different among survivors vs. non-survivors.” However, compared to baseline values, the final CI recorded was significantly increased in both survivors and non-survivors although final CI was not a significant predictor of in hospital mortality (Figure 1).

In order to observe the association between different RAP values and mortality, we ran multivariate logistic regressions adjusting for other significant predictors of mortality (age, number of pressors and inotropes received, and whether they received multiple devices during hospitalization). After adjustment, baseline RAP was no longer significantly associated with mortality in the overall cohort, while 24 h (OR 1.26 95% CI: 1.1–1.5) and final RAP (OR 1.3 95% CI: 1.1–1.6) remained statistically significant.

## Discussion

Our central findings are that persistently elevated RA pressure is significantly associated with worse outcomes and that reduced RA pressure within the first 24 h of treatment was an independent predictor of mortality. To our knowledge this is the first study to evaluate temporal changes in hemodynamics and an association with outcomes in patients receiving AMCS for cardiogenic shock. These observations suggest that management algorithms should focus not only in improving systemic blood pressure and cardiac output, but also on early and rapid decongestion. These observations may inform the development of future clinical trials and registries for cardiogenic shock.

Recently, the Society for Cardiovascular Angiography and Interventions (SCAI) staging scheme for CS severity identified 5 stages of shock defined by clinical, hemodynamic and metabolic variables. Several recent reports have validated the potential utility of this staging approach (9–11). Among the variables listed, central venous pressure <10 mmHg identifies Stage A patients who are at risk of developing CS. The remaining stages employ a RA:PCWP ratio of >0.8 to identify further deterioration. However, to acquire these data an invasive pulmonary artery catheter is required. Our findings suggest that among patients with CS requiring AMCS, which are generally Stage C, D, or E patients, the simple identification of an elevated RA pressure and a low MAP have potentially strong prognostic implications. These data suggest that monitoring RA pressure alone may be sufficient in early stage patients, and if elevated then further invasive hemodynamic data are required to fully characterize the severity of CS. Future studies in larger cohorts of patients are required to confirm the clinical utility of an isolated RA pressure on clinical outcomes in CS.

Throughout a patient's hospitalization for CS many interventions including fluid resuscitation, mechanical ventilation, hemodialysis, initiation of vasopressors or inotropes and use of AMCS can influence venous congestion. Few reports have examined temporal changes in hemodynamic variables over the course of a patient's hospitalization. We observed that as baseline PCWP was elevated and CI was low before initiating AMCS for CS. Within the first 24 h of initiating AMCS, PCWP or CI were not significantly different between survivors and non-survivors. In contrast, RA pressure was elevated at baseline between survivors and non-survivors, but after initiation of AMCS, survivors showed a statistically significantly lower RA pressure compared to non-survivors. Although baseline RAP was not associated with higher mortality after adjusting for other variables, 24 h RAP and final RAP remained statistically significant. These findings suggest that timely venous decongestion may be an important aspect of CS management that improves survival. Whether specific methods used to reduce venous congestion are superior to others remains largely unexplored. These novel findings also provide important insight into CS by suggesting that venous congestion may worsen outcomes through several mechanisms: (1) causing right heart congestion, thereby leading to a shift in the interventricular septum and reducing LV capacitance and stroke volume; (2) elevated venous congestion contributes to worsening pulmonary congestion by decreasing pulmonary artery compliance; and (3) by causing worsening renal vein congestion, thereby reducing renal perfusion and glomerular filtration, which in turn worsens systemic congestion. With further study the importance of venous decongestion in CS may become a central aspect of patient management.

Limitations of the present study include the retrospective nature of the analysis, the small cohort with available clinical data and hemodynamics from admission to discharge which also limited our ability to evaluate the impact of changes in RAP based on etiology of cardiogenic shock (ischemic cardiomyopathy vs. non ischemic cardiomyopathy), SCAI stages, or MCS platform (ECMO vs. Impella vs. IABP). We are planning to replicate this analysis on a larger, multicenter database which will help address those limitations. Furthermore, our analysis included only patients receiving AMCS. This was intentional to identify critically ill shock patients. Other important hemodynamic parameters associated with outcomes in CS were not included in this analysis, like Cardiac Power Output (CPO). However, future studies will need to study temporal changes in hemodynamics across all stages of CS severity. Also, the inherent selection bias regarding device appropriation per patient. Finally, the small size of the study did not allow for a rigorous multivariate analysis.

In conclusion, we report a novel retrospective analysis of hemodynamic changes in patients with CS receiving AMCS. Our findings identify the potential importance of venous congestion as a prognostic marker of mortality and further that early decongestion or reduced RA pressure is associated with better survival in these critically ill CS patients. These observations suggest the need for further study in larger retrospective and prospective cohorts of patients with varying degrees of CS severity.

## Data Availability Statement

The raw data supporting the conclusions of this article will be made available by the authors, without undue reservation.

## Author Contributions

All authors contributed to the design and implementation of the research, to the analysis of the results, and to the writing of the manuscript.

## Conflict of Interest

NK has received research funding from Abbott; Abiomed Inc., Boston Scientific Inc.,Getinge; LivaNova; MD Start Inc., and preCARDIA Inc. The remaining authors declare that the research was conducted in the absence of any commercial or financial relationships that could be construed as a potential conflict of interest.
